# Supplementary material for: Growing Homophilic Networks Are Natural Navigable Small Worlds
Source: PLoS One. 2016 Jun 27;11(6):e0158162. doi: 10.1371/journal.pone.0158162 (PMC4922669; doi:10.1371/journal.pone.0158162)
Supplement: S1 File — Supplementary part of the paper that includes: comparison of the basic GH model to the scale-free networks from [16] (Figure A); plots of self-similarity in clustering coefficient distribution (Figure B); plots of average nearest neighbor degree (Figure C); plots of rich club coefficient (Figure D); pseudocode of the construction algorithm; pseudocode of the degree normalized distance algorithm; plots of stretch and upper limits of the greedy path in 1D (Figure E). (PDF) [file pone.0158162.s001.pdf]

Supporting Information for
"Growing homophilic networks are natural navigable small worlds"
by Yu. A. Malkov and A. Ponomarenko

1. Comparison of the basic GH model to the scale-free networks from ref. [1] (Boguna, M., Krioukov, D., & Claffy, K. C. (2009). *Nature Physics*, *5*(1), 74-80).

The scale-free networks from [1] were modeled with the parameters γ=5, α=2.5 as advised in the paper, for 1D uniformly distributed data in Euclidian distance. The characteristic scale parameter was adjusted to produce a success probability close to 0.92, resulting in an average node degree close to 12. The parameter *M* of the GH algorithm was set to 5, thus the network had an average degree of 10. The comparison was done only for the 1D case, because for the higher dimensions we were not able to simultaneously get high success probability and the low average degree for the scale-free networks from [1]. The maximum data size in the comparison was limited by the algorithm from [1] which had a higher $N^2 \ln(N)$ complexity to establish the scaling (compared to $\sim N\cdot\ln^2(N)$ complexity for the GH algorithm case). Both for the GH networks and for the scale-free networks the plots were smoothed over 8-12 trials. An averaged success probability of a greedy search does not decrease with the number of elements in the network for both models (see Fig A(b)).

Smoothed greedy search success probability scaling is plotted in Fig A(d) for a case of GH networks with higher dimensional data (d=4, 6). The plot shows almost no changes in the success probability as the networks grows by several orders of magnitude thus demonstrating the GH networks are also navigable by the definition from ref. [1]. This shows the existence of a new class of navigable models with exponential degree distribution. Navigability in this case was not expected from the results of ref. [1], where a scale-free degree distribution was required for the navigability.

While the considered scale-free networks offer significantly less hops (Fig A(c)), the number of distance computations for the scale-free network has a scaling close to $N^{1/1.5}$(Fig A(a)) which corresponds to a $N^{1/(\gamma-1)}$ scaling of the maximum degree in the network. The GH networks instead have a polylogarithmic scaling. So despite the small number of hops the greedy search can hardly be called local for the scale-free networks, especially in the case of the γ close to 2. The observed polynomial scaling should also be valid for hyperbolic geometric graphs[2] which share basic properties with networks, studied in ref. [1].

2. Self-similarity in clustering coefficient distribution

The plot in Fig B demonstrates a self-similar structure of the network's average clustering coefficient distribution, obtained using the same procedure as in [3]. All nodes with degree less than k_{thr} are removed from the network and degrees of the nodes are normalized by the mean degree. Finally, a distribution of normalized average clustering coefficient is calculated for different k_{thr}.

Alternating the k_{thr} in a wide range does not change the normalized average clustering coefficient distribution, which means that the network has a self-similar structure[3].

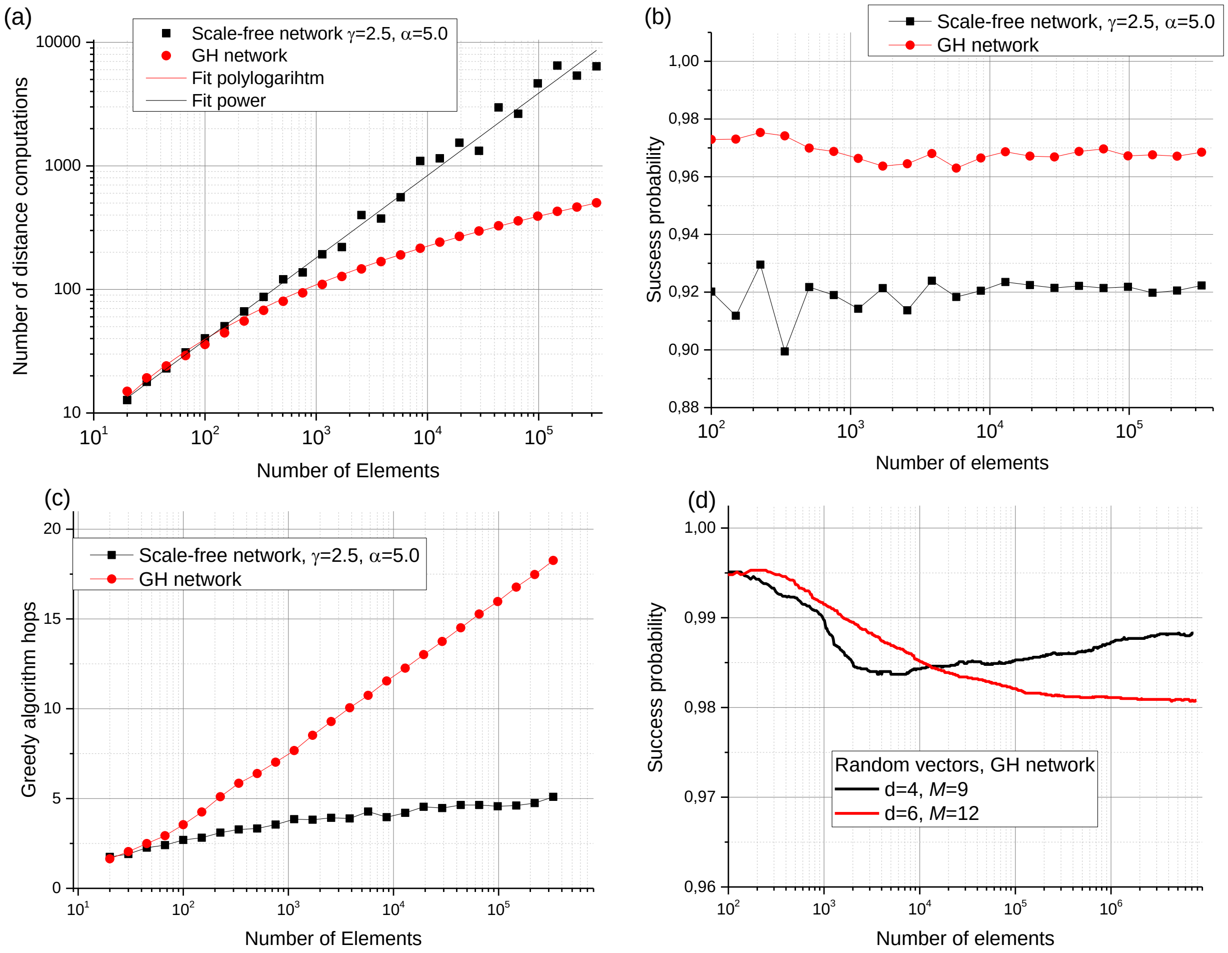

Fig A. Comparison of the GH algorithm to the navigable scale-free networks from [1] on 1D data (a-c). (a) Average number of distance computations during a greedy search. (b) Average success probability of a greedy search. (c) Average greedy algorithm hops.

(d) The scaling of greedy search success probability in GH networks on higher dimensional random data.

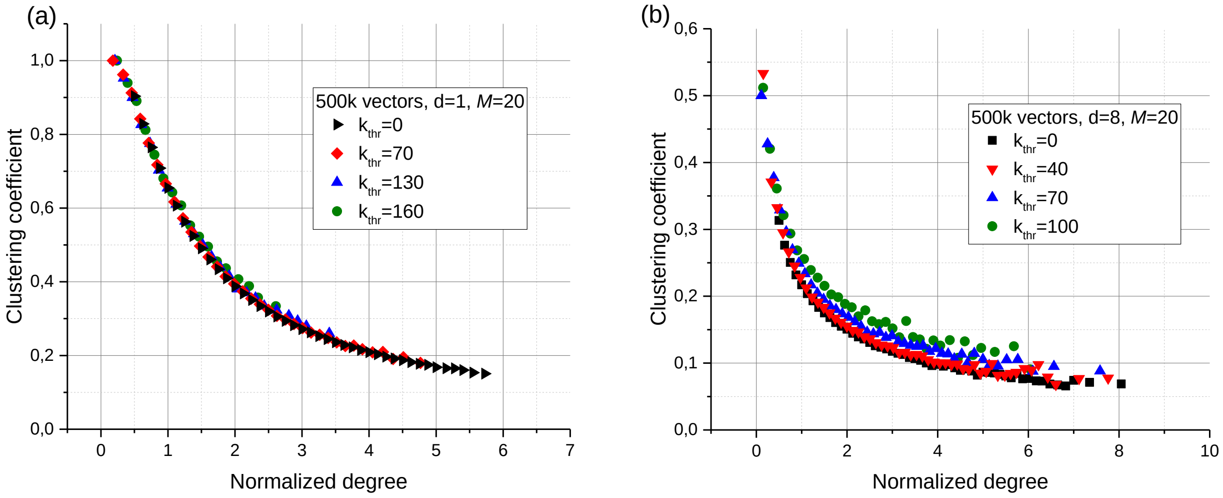

Fig. B. Normalized average clustering coefficient distribution in the network for different values of k_{thr} and dimensionality.

3. Average nearest neighbor degree

The plot in Fig C shows average nearest neighbor degree distribution demonstrating that GH networks have assortative degree mixing.

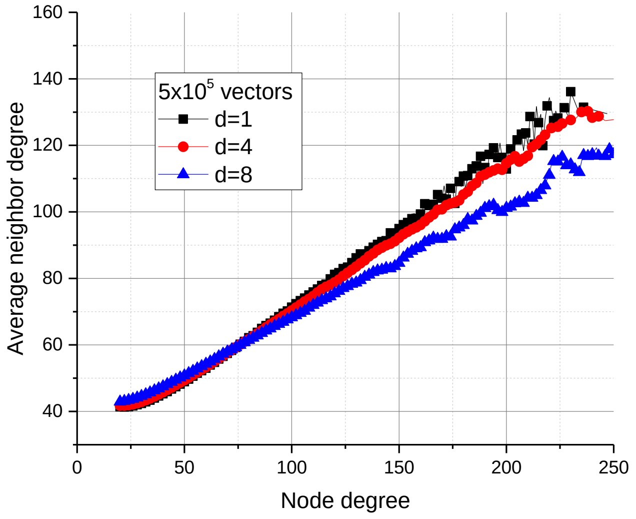

Fig. C. Nearest neighbors degree distribution for vectors with different dimensionality.

4. Rich club coefficient

Figure D shows an exponential rise of the rich club coefficient $\phi(k) = 2E_{>k} / (N_{>k}(N_{>k} - 1))$ [4] together with a rich club coefficient of a random network with the same degree distribution (for the case of d=8), which demonstrates that GH networks have rich clubs.

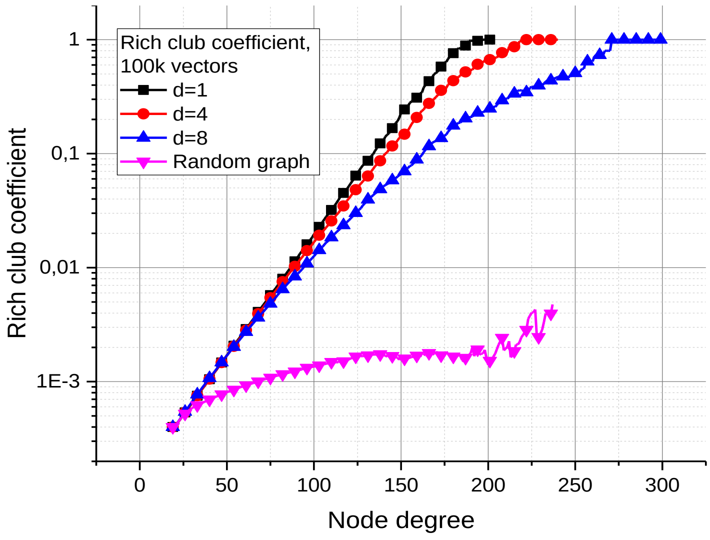

Fig. D. Rich club coefficient of GH networks for vector data of different dimensionality

5. Pseudocode of the construction algorithm

During the insertion of a new element q to the network, it is connected to M approximate closest elements from the existing network. To obtain approximate *M* nearest neighbors, a dynamic list of *M* closest of the found elements (initially filled with a random enter point node) was kept during the

search. The list was updated at each step by evaluating the neighborhood of the closest previously non-evaluated element in the list until the neighborhood of every element from the list was evaluated. For *M*=1, this method is equivalent to a basic greedy search. The best *M* results from `NumTrials` trials were used as approximate closest elements. The number of trials was adjusted so that the recall (the ratio between the found and the true *M* nearest neighbors) was higher than 0.95, producing results almost indistinguishable from what you get from the exact search (typically, `NumTrials` was set to 10-20 in the experiments).

```
Alg. 1
K-NNSearch(object q, integer: NumTrials, M)
Set [object] tempRes, candidates, visitedSet, result
for (i ← 0; i < NumTrials; i++) do:
     put random entry point in candidates
     tempRes ← null
     repeat:
            get element c closest from candidates to q
            remove c from candidates
            //check stop condition:
            if c is further than M-th element from result
                          than break repeat
            //update list of candidates:
            for every element e from friends of c do:
                   if e is not in visitedSet than
                          add e to visitedSet, candidates, tempRes

     end repeat
     //aggregate the results:
     add objects from tempRes to result
end for
return closest M elements to q from result
```

6. Pseudocode of the degree normalized distance algorithm

In the experiments with preferential attachment (PA) and for the example in Fig 3 in the manuscript we used the modified distance function between a target search element and an element from the network. The distance is computed by dividing the standard L2 distance by a power function of a network element degree, thus making high degree nodes effectively “closer” to the target than they were in the original Euclid space. The power in the degree function is set to 1/d, where d is the dimensionality of the vector space. The preference to high degree is set to be saturated at a constant k_c: if the element degree is higher than k_c, than the L2 distance is just divided by the power function of the k_c. Note that setting k_c less than M in GH networks leads to complete absence of the high degree preference.

Mentioned modified distance was used to find the approximate connections for the case of GH with PA. I.e. instead of just connecting a new element to approximate closest elements by the L2 distance, the new element is rather connected to the elements which minimize the presented effective distance. The degree normalized distance also was used during greedy searches to get the short path for the experiment presented in Fig 3.

```
Alg. 2
degree_normalized distance(object query, object networkObject, integer: d ,k_c):
if networkObject.degree>k_c
     return L2_distance(query, networkObject)/(k_c)^(1/d)
else
     return L2_distance(query, networkObject)/(networkObject.degree)^(1/d)
```

7. Stretch and upper limits of the greedy path in 1D

The plots of stretch (the ratio between the greedy path length and the shortest path length) are presented in Fig E(a) for two variants of the greedy algorithm for d=1, M=9. At small dataset size the stretch is close to 1 due to cliquishness of the GH network at low dataset sizes. For 1D vectors with the rise of the dataset size the stretch increases and later saturates at the value close to 2, in agreement with the prediction from the consideration of the greedy algorithm hops upper bound. When using the degree normalized search strategy described in the previous section the stretch stays close to one, meaning the deduced paths are almost as short as the shortest.

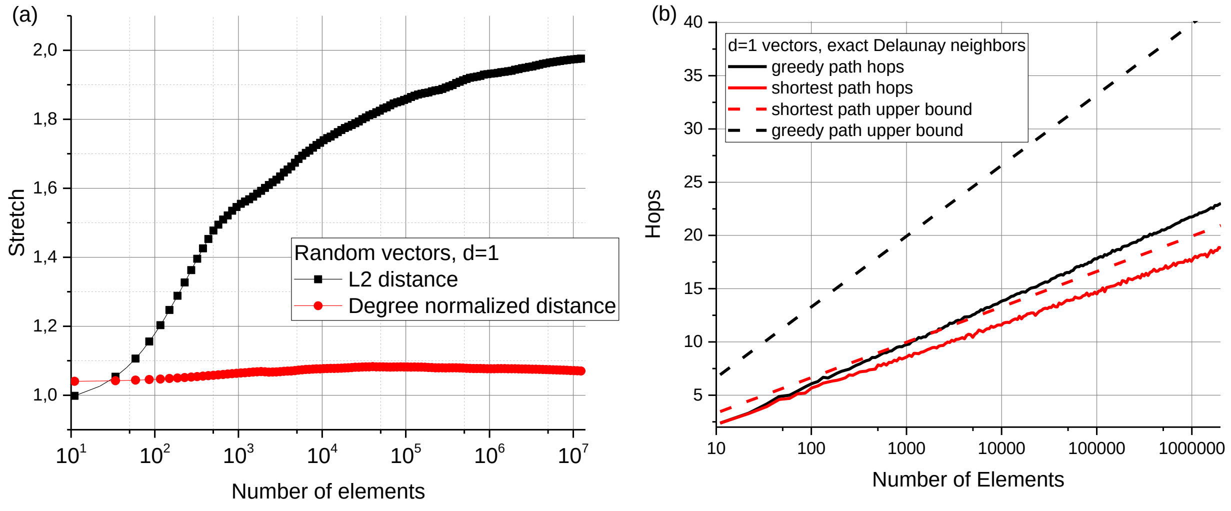

Fig. E. (a) Stretch (the ratio between the greedy path length and the short path length) for two variants of the greedy algorithm for d=1. (b) The greedy path hop number and shortest path length together with the upper bounds for the special case of connection to exact Delaunay neighbors in 1D.

The upper limit of the number of greedy hops and the shortest path length are depicted in Fig E(b) together with the evaluated number of the greedy hops and the shortest path length for d=1 with connection to the exact Delaunay neighbors (the closest neighbor in each of two directions). Connection to exact Delaunay neighbors leads to perfect success probability, minimal number of edges and strong correlation between the neighbors distance and the time of element addition. Mentioned correlation can explain that the greedy paths are almost as short as the global shortest paths for this case. Note that for the case of larger M (Fig E(a)) the correlation between the neighbors distance and the time of element addition should be much weaker due to the overlap of the distance spread between the connections of consecutive generations.

References

1. Boguna M, Krioukov D, Claffy KC. Navigability of complex networks. Nature Physics. 2009;5(1):74-80.
2. Krioukov D, Papadopoulos F, Kitsak M, Vahdat A, Boguná M. Hyperbolic geometry of complex networks. Physical Review E. 2010;82(3):036106.
3. Serrano MA, Krioukov D, Boguná M. Self-similarity of complex networks and hidden metric spaces. Physical Review Letters. 2008;100(7):078701.
4. Colizza V, Flammini A, Serrano MA, Vespignani A. Detecting rich-club ordering in complex networks. Nature Physics. 2006;2(2):110-5.
